# Research progress of SWI/SNF complex in breast cancer

**DOI:** 10.1186/s13072-024-00531-z

**Published:** 2024-02-17

**Authors:** Kexuan Li, Baocai Wang, Haolin Hu

**Affiliations:** 1 School of Medicine, Southeast University, Nanjing, 210009 Jiangsu China; 2 Department of Surgery, TUM School of Medicine, Klinikum rechts der Isar, Technical University of Munich, 81675 Munich, Germany; 3 Breast Center, Zhongda Hospital, School of Medicine, Southeast University, 87 Dingjiaqiao Road, Nanjing, 210009 Jiangsu China

**Keywords:** Breast cancer, Epigenetics, SWI/SNF, Chromatin remodeling

## Abstract

In the past decade, numerous epigenetic mechanisms have been discovered to be associated with cancer. The mammalian SWI/SNF complex is an ATP-dependent chromatin remodeling complex whose mutations are associated with various malignancies including breast cancer. As the SWI/SNF complex has become one of the most commonly mutated complexes in cancer, targeting epigenetic mutations acquired during breast cancer progress is a potential means of improving clinical efficacy in treatment strategies. This article reviews the composition of the SWI/SNF complex, its main roles and research progress in breast cancer, and links these findings to the latest discoveries in cancer epigenomics to discuss the potential mechanisms and therapeutic potential of SWI/SNF in breast cancer.

## **Background**

Among women, breast cancer has become the most commonly diagnosed cancer and is currently a leading cause of cancer worldwide [1]. Despite remaining the leading cause of cancer deaths among women, the mortality rate of breast cancer has declined in the past decade. This can mainly be attributed to detecting breast cancer earlier through screening, as well as to improved understanding and treatment of the disease. Molecular subtypes of breast cancer are of great significance for individualized treatment of patients. Epigenetic abnormalities contribute to the development of cancer, and cancer cells often change the levels or activity of epigenetic regulatory proteins [2]. Therefore, continuing to explore effective molecular markers in breast cancer is of great importance for clinical treatment. The SWI/SNF chromatin remodeling complex has been found to be one of the most widely mutated protein complexes in human cancers. This complex uses the energy generated by ATP hydrolysis to mobilize nucleosomes, regulating chromatin structure [3]. Previous studies have found that approximately 30% of breast tumors have genetic alterations in one or more SWI/SNF subunits, with gene amplification being the most common. These gain and loss of function mutations are associated with cancer initiation and progression in various ways [4]. Therefore, this article reviews the research progress of SWI/SNF complex subunits in breast cancer in recent years.

### Introduction to the SWI/SNF family

**Fig. 1:**
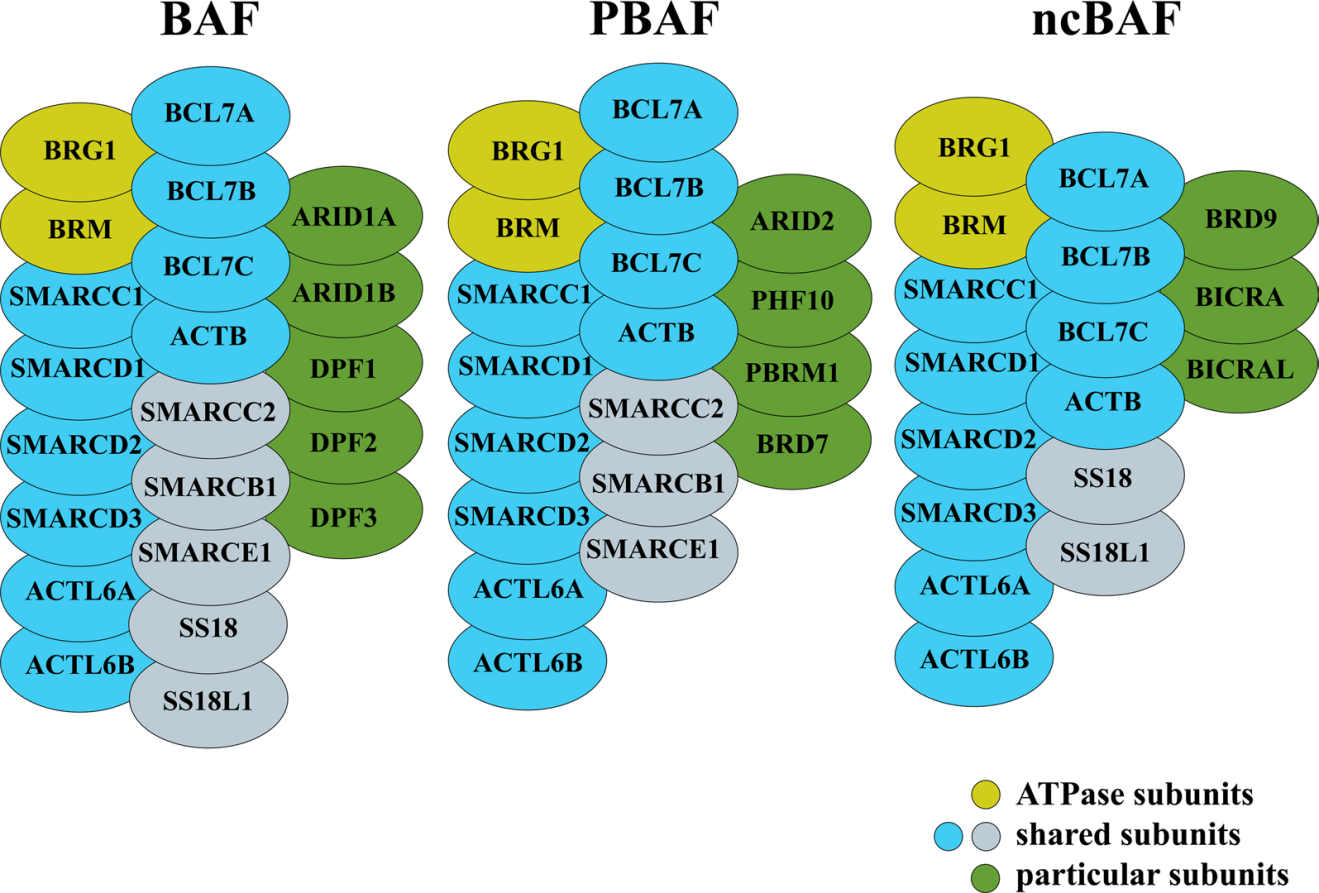
The three forms of SWI/SNF complex and subunits of composition: The SWI/SNF complex is composed of products of a total of 29 genes, forming three final forms of complexes containing different variations: BAF, PBAF and ncBAF, each complex is composed of ATPase subunit(yellow), shared subunits(blue and grey) and particular subunits(green)

The SWI/SNF complex is composed of three types of subunits: the ATPase, core subunits, and auxiliary subunits (Fig. 1) [5, 6]. Although the exact mechanism by which the SWI/SNF complex modifies chromatin structure is not fully understood, current knowledge suggests that its main mechanism involves ATPase-dependent disruption of histone-DNA binding and the resulting “sliding” of nucleosomes. The SWI/SNF complex remodels nucleosome structure, using the energy of ATP, to mobilize nucleosomes by sliding and catalyzing the eviction of histone octamers [7]. These complexes play important roles in gene expression regulation and maintaining stem cell pluripotency [8]. Each SWI/SNF complex contains only one of two ATPases, BRM (Brahma) or BRG1 (Brahma-Related Gene 1). The SWI/SNF complex is composed of products of a total of 29 genes, forming three final forms of complexes containing different variations: BAF (BRG1/BRM-associated factor), PBAF (Polybromo-associated BAF complex), and newly defined ncBAF (non-canonical BAF) [9–11]. Each complex isoform has 10–12 proteins as core and auxiliary subunits. The ARID1A (BAF250a) and ARID1B (BAF250b) subunits exclude each other and exist only in the BAF complex. BAF180, BAF200, and BRD7 exist only in the PBAF complex [12]. The subunits specific to the ncBAF complex mainly include GLTSCR1/GLTSCR1L and BRD9, which mainly regulate preserved non-fusion sites and maintain the gene expression of preserved mSWI/SNF (mammalian SWI/SNF) sites [9, 10].

Chromatin remodeling complexes participate in the dynamic transcriptional regulation of many genes. Although SWI/SNF performs biochemical functions different from typical sequence-specific transcription factors (they act as master regulators during development and control the expression of numerous targets), it cooperates with or even control these factors to regulate overlapping downstream targets. Therefore, a single mutation that causes SWI/SNF inactivation or activation of a “master” transcription factor may lead to changes in the regulation of many downstream genetic pathways [13].

As tumor suppressor genes are defined as “guardians” that maintain genomic integrity and regulate cell proliferation, cell cycle, or promote apoptosis [8], recent studies have found that the development of tumors depends on both epigenetic and genomic changes [14, 15]. Chromatin structure is regulated by two classes of cooperatively acting complexes: the first class covalently modifies histone tails, and the second class reshapes nucleosomes in an ATP-dependent manner. In ATP-dependent chromatin remodeling, the SWI/SNF complex, which bears 9 ~ 12 subunits individually and has ATP-dependent nucleosome remodeling activity, is the most common dysregulated complex in cancer [16, 17]. Mammalian SWI/SNF enzyme function is highly dependent on the environment, and changes in relative subunit concentrations can lead to pathological gene activity through aberrant gene (in) activation, which can have negative or positive effects on transcriptional activity [18]. Therefore, overexpression of SWI/SNF subunits may similarly lead to the development or acceleration of cancer [19]. Regarding the epigenetic tumor suppressor function, many studies have shown that SWI/SNF inactivation leads to increased sensitivity to DNA damage, suggesting that these complexes play an important role in DNA damage response [20, 21]. ARID1A has been proved to directly participate in DNA repair processes, such as promoting DNA end resection [22]. Additionally, several studies have suggested that ARID1A is associated with transcriptional regulation by inducing nuclear hormone and expression of cell cycle regulators. Notabaly, ARID1A mutations are common in hormone-responsive cancers such as breast and ovarian cancer [21, 23–25].

### The role of SWI/SNF complex in breast cancer

The concept of precision oncology is based on the assumption that understanding the genomic basis of a patient’s cancer will guide the selection of potentially effective targeted therapies [26]. Known and relatively common breast cancer gene mutations, such as TP53, PIK3CA, and GATA3, are typically found in primary and recurrent specimens. In contrast, mutations in low-frequency cancer genes are typically only found in cancer relapse. This pattern is particularly significant for genes involved in SWI/SNF signaling, such as ARID1A, ARID1B, and ARID2, which are typically wild-type in primary lesions but inactivated in recurrent lesions. The following sections of this article will provide an overview of the specific research progress on SWI/SNF complex-related subunits in breast cancer.

## The ARID1A and ARID1B BAF-specific subunits in breast cancer

### ARID1A

A vital role is performed by ARID1A under regular circumstances, ensuring genomic stability and addressing DNA damage [27]. Although ARID1A is not a core subunit of the SWI/SNF complex, it is the most common SWI/SNF mutation component in breast cancer [28]. As ARID1A functions in cell cycle inhibition, based on the observed loss-of-function mutation spectrum in various cancers, it is speculated to be a tumor suppressor. It has been found that acquisition of ARID1A function can be promoted by enhancing oxidative stress [29, 30]. Conversely, the loss of ARID1A during late-stage tumor growth hinders the transcription of genes linked to migration, invasion, and metastasis. Further research is necessary to elucidate the reasons behind the observed variations in the expression of this biomarker [30].

**Fig. 2:**
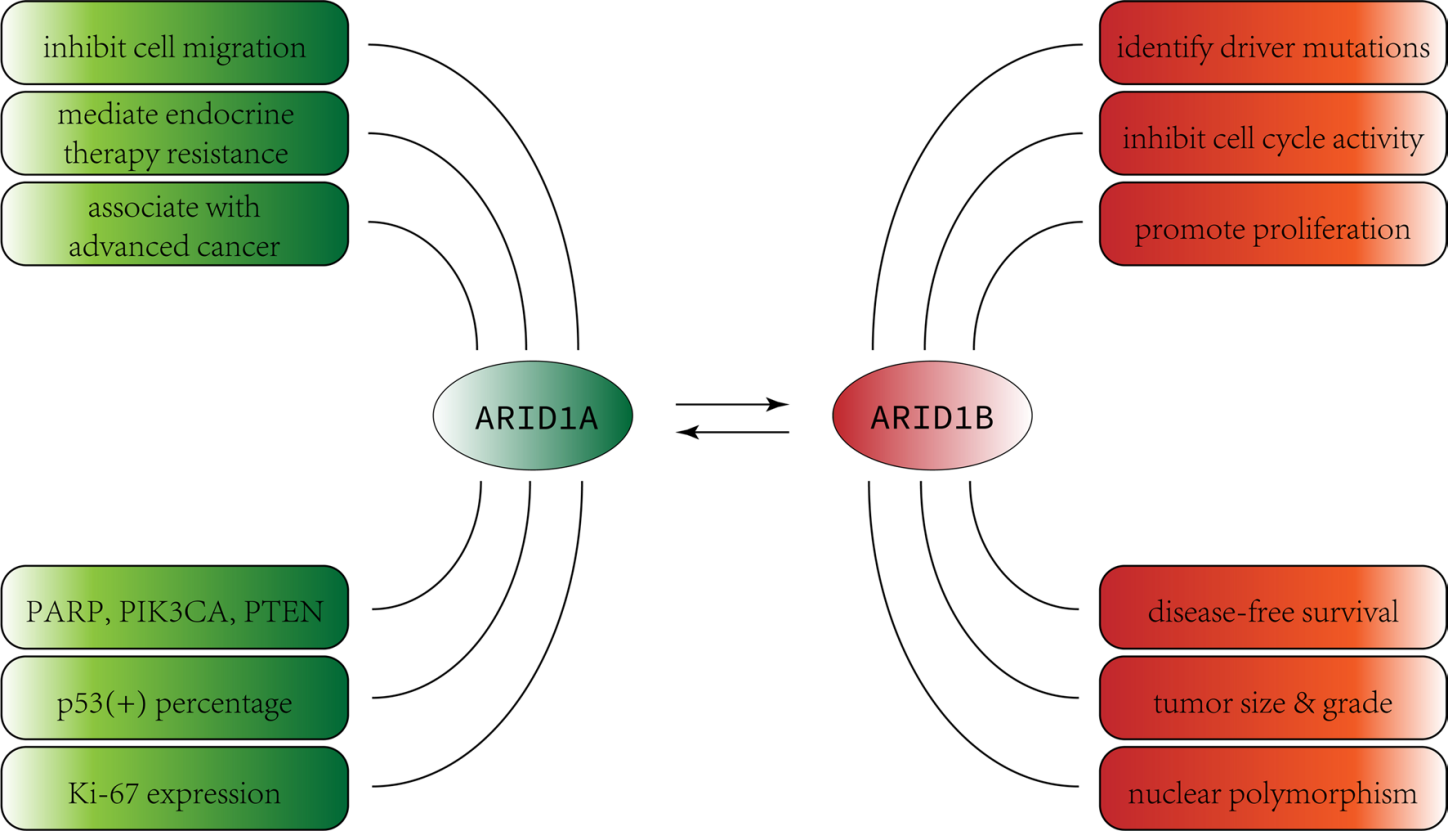
Influences ofARID1A and ARID1B on breast cancer: As is illustrated in this figure, ARID1A can affect breast cancer by (**a**) inhibiting cell migration, (**b**) mediating endocrine therapy resistance and it has association with metastatic breast cancer. Its expression tend to impact the function of PARP, PIK3CA, PTEN, P53(+) percentage, Ki-67 expression and so on. For ARID1B, its roles include identifying driver mutations, inhibiting cell cycle activity, promoting proliferation, e.t.c. The level of ARID1B expression is associated with the disease-free survival, tumor size and grade, as well as the nuclear polymorphism

After migration assays, it was found that ARID1A can effectively inhibit cell migration in multiple breast cancer cell lines (Fig. 2). These results strongly suggest that in vitro, ARID1A is a tumor suppressor gene in breast cancer cells [31]. Loss of ARID1A in the process of carcinogenesis may make early cancer cells prone to genomic instability. Therefore, increasing ARID1A levels and SWI/SNF complex activity in normal breast epithelium may be beneficial in inhibiting the formation of cancer cells. With CRISPR/Cas9 technology, scientists have found that breast cancer cells with ARID1A knockout had increased proliferation under estrogen deprivation conditions, compared to controls [32]. Several studies have shown a correlation between loss-of-function mutations in ARID1A and the presence of activating mutations in PIK3CA, loss of PTEN expression [33], and loss of p53 function [34, 35]. The inhibition of PARP1, HDAC2/6, Aurora kinase A, CDK4/6, or PI3K/Akt has led to many synthetic lethality (the creation of a lethal phenotype from the combined effects of mutations in two or more genes) treatment opportunities based on ARID1A mutations and mismatch repair defects [36–38].

ARID1A mutations are also considered a mediator of endocrine therapy resistance in metastatic ER-positive breast cancer [28]. Recent analyses suggest that ARID1A changes are more abundant in the metastatic environment after endocrine therapy than in untreated primary tumors. The impact of ARID1A loss on gene expression was assessed using RNA sequencing (RNA-seq) analysis. The results revealed a significant effect and emphasizing the critical role of chromatin controlled by ARID1A in gene expression in ER-positive breast cancer. Observational results also suggest that ER binding to chromatin depends on ARID1A. Consequently, the loss of ARID1A affects estrogen-mediated transcription, with over 40% of estrogen-regulated genes relying on ARID1A for regulation. After the loss of ARID1A, the expression of ER target genes is significantly downregulated. Clinical genomic studies have further demonstrated that patients with ARID1A alterations exhibit a poorer response to ER degradation products, indicating a potential weakening of ER dependency in these patients.

It has been proven that ARID1A plays an important role in chromatin reprogramming, and inactivated ARID1A mutations occur more frequently in metastatic tumors and tumors with hormone therapy progression. This may give rise to the long-term selective inhibition of ER, which results in cells with acquired inactivated ARID1A mutations, becoming independent of ER therapy. Further research is needed to determine whether epigenetic regulation can reverse or delay lineage transitions, thereby prolonging the response to endocrine therapy [32]. Clinical pathological analysis shows that low ARID1A mRNA expression in breast cancer is associated with advanced tumors, increased P53(+) percentage, and high Ki-67 expression, and 78% of triple-negative breast cancers exhibit low ARID1A expression [39].

Dysregulated cell proliferation is a key hallmark of cancer, and targeting cell proliferation signaling is a widely used strategy in cancer treatment. Therapeutic strategies have been reported for ARID1A-mutant breast cancer based on synthetic lethality and cell dependency, such as some PARP, EZH2, and BRD4 inhibitors. Currently, CDK4/6 inhibitors have shown promising potential as a new strategy for treating ARID1A-mutant metastatic breast cancer. Additionally, research has revealed that cells with reduced ARID1A expression have a selective decrease in survival when treated with PARP inhibitors. The absence of ARID1A may render cancer cells more sensitive to PARP inhibition through specific pathways. PARP inhibitors also demonstrate targeted effectiveness against ARID1A-deficient cells [40]. Further mechanistic studies and clinical trials are needed to determine the efficacy of cancer targets driven by ARID1A mutations [41].

## ARID1B

ARID1B plays an important role in mammalian development by regulating the cell cycle during differentiation [42]. Studies using comprehensive analysis of cancer genome sequencing have identified loss-of-function mutations in either ARID1A and/or ARID1B in various cells, suggesting that they may encode products related to tumor development [43]. An in vitro study showed that ARID1B is a specific determinant of the SWI/SNF complex, has a broad role in promoting proliferation, and also plays a significant role in inhibiting cell cycle activity, making it an attractive therapeutic target for cancer treatment [19, 42]. There is experiments proving that cell proliferation and cell cycle progression are disturbed in embryonic stem cells (ES cells) deficient in ARID1B, which may have something to do with the markedly reduction of the expression of many pluripotency genes. Data suggest that ES cells with low expression of ARID1B are deficient in maintaining undifferentiated state [44]. Compared with this, another study shows that BAF250a is critical for the maintenance of ES cell self-renewal, and for lineage-specific differentiation of ES cells in vitro [45]. A previous study demonstrated that ARID1B exhibits specific vulnerability in human cancers with ARID1A mutations, and that ARID1A deficiency may make cancer cells more dependent on ARID1B. Depletion of ARID1B also disrupts the stability of the SWI/SNF complex and inhibits the proliferation of ARID1A-deficient breast cancer [41, 46]. These two homologs are mutually exclusive, and any one BAF complex contains only one of them [16, 47]. Loss of ARID1A cannot be compensated by increasing ARID1B expression, and in most tumor samples, the expression of ARID1B is also lower than that of ARID1A [48], suggesting that ARID1B is necessary for promoting tumor development in ARID1A mutations. Given its proliferative function, ARID1B may have an opposite effect in tumors caused by ARID1A mutations [19].

In breast cancer, ARID1B is identified as one of the driver mutations that give cancer cells a clonal selective advantage [49]. Univariate statistical analysis showed that high expression of ARID1B is closely related to the age of breast cancer patients, tumor size, histological tumor grade, and nuclear polymorphism. Multivariate data analysis further suggests that ARID1B expression level is an independent predictive factor for tumor size, tumor grade, and nuclear polymorphism. In addition, analysis has shown that the level of ARID1B expression is associated with the disease-free survival of breast cancer patients, and that the 5-year survival rate of ARID1B-positive patients is significantly lower than that of ARID1B-negative patients [19]. Therefore, high expression of ARID1B may contribute to the occurrence of breast cancer. Current research also indicates that ARID1B is highly expressed in triple-negative breast cancer subtypes compared to other molecular subtypes of breast cancer [43].

In summary, ARID1B may be a prognostic and predictive biomarker for breast cancer and an effective therapeutic target for its treatment.

## The PBRM1 and ARID2 PBAF subunits in breast cancer

### PBRM1

PBRM1 is located on chromosome 3p21 and is a component of the SWI/SNF complex in mammalian cells, encoding the BRG1-associated factor 180 protein. The protein encoded by this gene participates in DNA replication, transcription, repair, and regulates cell proliferation and differentiation. Due to its ability to regulate p21 expression under different environmental stimuli (such as TGF-β treatment or DNA damage), BAF180 is crucial for regulating the cell cycle and plays a vital role in preventing tumor development by promoting centromere cohesion and ensuring genomic stability [50]. Studies have shown that PBRM1 not only plays a critical role in preventing tumor development, but also participates in regulating genes implicated in the DNA damage response and maintaining redox equilibrium [51, 52]. PBRM1 binds to the p21 promoter and regulates baseline and signal-dependent p21 transcription, thereby inhibiting tumor development in breast cancer cells, indicating that PBRM1 is a tumor suppressor gene mutated in breast cancer [53]. PBRM1 is an essential factor that independently impacts the prognosis of breast cancer patients. Its expression is strongly correlated with the clinical stage and lymph node status of breast cancer patients, serving as a significant indicator for overall survival and recurrence-free survival in these patients. Compared to matched non-cancerous tissues, the expression of PBRM1 is significantly decreased in breast cancer tissues, and its low expression is associated with poor clinical outcomes in breast cancer patients, further indicating its tumor suppressor function [54]. In addition, PBRM1 also affects the anti-tumor immune response [55], especially in preclinical cancer models by mediating resistance to T-cell-dependent killing [56, 57]. As a tumor suppressor gene, PBRM1 may be involved in the occurrence and development of breast cancer and is a valuable prognostic indicator for breast cancer patients, further research is needed to explore other potential molecular mechanisms [54].

### ARID2

Similar to PBRM1, ARID2 (BAF200) encodes a subunit that is only present in the PBAF complex. ARID2 is not a homolog of ARID1A/ ARID1B, although it shares a common AT-rich interaction domain with these three subunits and some common structural similarities, but it is mutually exclusive with ARID1A/ ARID1B. ARID2 frequently undergoes mutations in other malignant tumors [58]. Research indicates that there is a frequent occurrence of low expression of ARID2 in non-luminal breast cancer subtypes. Additionally, this lowered expression is a predictor of poor survival in ER-positive breast cancer patients [59]. So far, there are few reports on the relationship between ARID2 and breast cancer, and further research is needed to determine the role and mechanism of ARID2 in breast cancer.

## BRG1&BRM

The SWI/SNF complex always contains a catalytic subunit, either BRM or BRG1. BRM and BRG1 are mutually exclusive ATPase subunits, and studies have identified BRM as an effective synthetic lethal target for BRG1-deficient cancers, and vice versa [37, 60, 61]. The SWI/SNF complex containing BRG1 plays a critical role in regulating cell proliferation, lineage specification, and maintaining pluripotency in early embryonic development. Interestingly, BRG1’s function varies depending on the type of cancer, as it can either exhibit tumor-suppressive or tumor-promoting properties [62, 63]. Many reports indicate that BRG1 promotes autophagy and hinders apoptosis. These processes aid cancer development and impede treatment. BRG1 collaborates with cancer-specific proteins to block apoptosis, and reducing BRG1 could facilitate alternative treatments like chemotherapy [64]. Studies have shown that both BRG1 and BRM are highly expressed in primary breast cancer compared to normal breast tissue and are necessary for in vitro and in vivo cancer cell proliferation [63, 65]. Therefore, BRG1 and BRM may be druggable targets in breast cancer, and the mechanism by which BRG1 expression increases cancer progression may be through promoting cell growth, migration, and invasion [65]. BRG1 can bind to the promoter of overexpressed genes in primary breast cancer cell lines and directly activate their transcription. BRG1 complexes formed at these sites interact with EP300 and PARP1 to guide breast cancer cell proliferation and transcription of DNA repair genes [66–68].

**Fig. 3:**
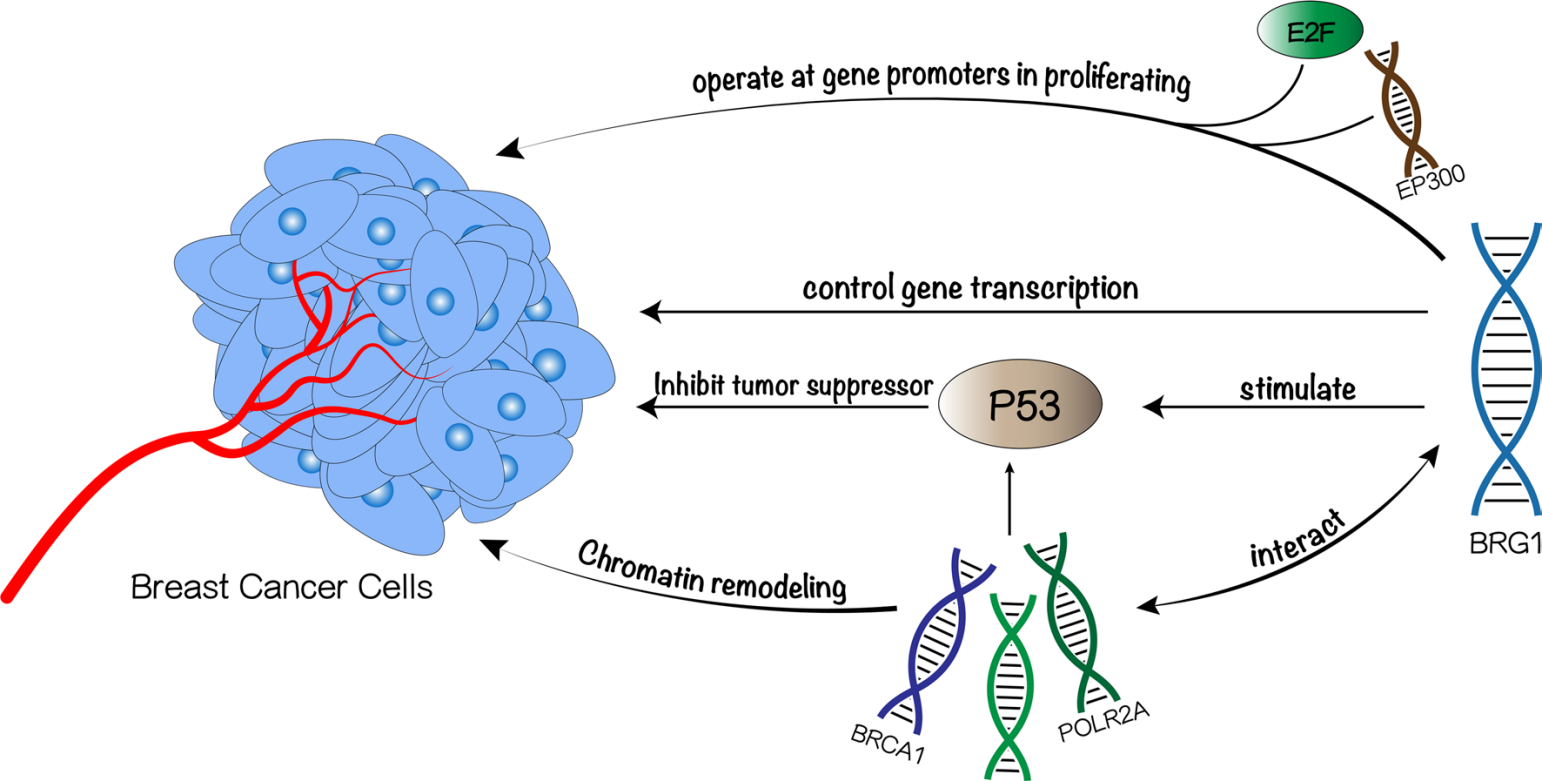
Different mechanisms of BRG1 promoting breast cancer cell proliferation: This figure lists some of the mechanisms that BRG1 promote breast cancer cell proliferation, BRG1 can: (1) operate with E2F transcription factors at gene promoters in proliferation in breast cancer cells, (2) controlling gene transcription, (3) stimulate transcriptional activity of the P53 protein and subsequently inhibiting the activity of tumor suppressor, (4) interact with BRCA1 to remodel chromatin and control the activity of p53 protein

In different types of breast cancer, BRG1 may promote cell proliferation through various mechanisms (as shown in Fig. 3), such as controlling gene transcription by promoting expression of genes responsible for cell cycle progression, and operating at gene promoters in association with E2F transcription factors. In addition, BRG1 directly interacts with BRCA1 tumor suppressor and subsequently stimulates transcriptional activity of the P53 protein, and has been documented to regulate cell proliferation by cooperating with P53 and then Inhibiting the activity of tumor suppressor [65–67]. In triple-negative breast cancer, BRG1 first promotes lipogenesis (especially fatty acid synthesis) to support cell proliferation. Even if exogenous fatty acids are available, tumor cells often use de novo fatty acid synthesis pathways, and key enzymes involved in fatty acid and lipid synthesis are frequently overexpressed in breast cancer. Knocking out BRG1 in triple-negative breast cancer cells significantly reduces de novo lipogenesis, which is associated with decreased cell proliferation. Additionally, BRG1 upregulates the expression of enzymes responsible for fatty acid and lipid biosynthesis and may upregulate them directly [69]. However, the universality of these findings in other types of breast cancer requires further investigation [18]. Given that BRG1 and BRM expression levels are negatively correlated with breast cancer patient prognosis, knocking out BRG1 or BRM may delay or weaken tumor initiation. Knocking out BRG1 or BRM leads to inhibited colony formation and xenograft formation. This suggests that BRG1 and BRM ATPases potentially promote breast cancer cell proliferation. By knocking out BRG1 and/or BRM, breast cancer cell proliferation can be reduced due to a decrease in the rate of cell cycle progression.

Additionally, evidence suggests that the effects of BRG1 and BRM on cell proliferation are partly mediated through independent mechanisms. Combined knockdown of BRG1 and BRM has an additive effect on cell proliferation, while individual CRISPR/Cas9-mediated knockdown of BRG1 or BRM reduces cell viability, indicating that BRG1 and BRM have at least partially non-overlapping roles in promoting breast cancer cell proliferation [63]. BRG1 knockout in mammary epithelial cells slows down the rate of cell proliferation, rather than inducing a cell type with more invasive features lacking tumor suppressors. Knockdown of BRG1 in breast cancer cells similarly leads to reduced proliferation rates in vitro and in situ xenografts [63, 65], while complete knockout of BRG1 via CRISPR/Cas9 technology results in cell death, confirming the requirement of BRG1 for breast cancer cell survival [63].

Studies have demonstrated that knockout of either ATPase reduces tumor formation and growth in xenografts and slows down cell proliferation in vitro in triple-negative breast cancer cells. These data suggest that both BRG1 and BRM are necessary to promote breast cancer cell proliferation in triple-negative breast cancer cell lines, and knockdown of both ATPases can precisely reduce cell proliferation by decreasing the rate of cell cycle progression [63]. Additionally, drug treatment increases the binding of BRG1 to drug transporter promoters. Recent reports show that BRG1 knockout increases sensitivity to a class of chemotherapy drugs currently used in clinical settings [70], such as 5-FU and paclitaxel, inducing BRG1-dependent drug transporter gene expression leading to increased intracellular drug concentrations. Some of these results have been attributed to BRG1-dependent induction of ABC transporters [70, 71]. Knockout of BRG1 eliminates transporter induction and, more importantly, increases intracellular drug concentrations, thus increasing chemotherapy sensitivity [18]. Recent data also suggest that the lack of BRG1 activity may make cancer cells susceptible to drugs by inhibiting drug efflux and weakening DNA damage elimination. Attempts to inhibit BRG1 and/or BRM may be particularly important in the treatment of triple-negative or ER/PR-negative and HER2-low to normal breast cancer, especially in cases where targeted therapy is not feasible and patients can only benefit from low-targeted cytotoxic drugs. These findings suggest potential future applications of BRG1 and/or BRM inhibitors or antagonists in breast cancer treatment [67].

## Conclusion

The SWI/SNF complex plays a critical role in the regulation of gene expression and chromatin remodeling. In breast cancer, studies have shown that alterations in the SWI/SNF complex components, including mutations and aberrant expression levels, are frequently observed. These alterations can lead to dysregulation of key genes involved in tumor suppression, DNA repair, and cell cycle control, contributing to the development and progression of breast cancer. Given the vast involvement of the complex and its associates in diverse physiological pathways, through direct and indirect means, the incidence of SWI/SNF’s influence on cancers might be considerably higher [72]. Furthermore, extensive research has linked the SWI/SNF complex to drug resistance and metastasis in breast cancer. Deciphering the intricate relationship between breast cancer and the SWI/SNF complex not only enhances our understanding of the molecular mechanisms driving this disease but also holds promise for the advancement of targeted therapeutic approaches.

Cancer is a progression of many changes in cellular states, with one or more programmed genetic alterations driving increased proliferation or a cancer metabolic phenotype. Recent technological advancements have paved the way for the study of single-cell epigenetics, with single-cell CHIP-seq used to profile active and inhibitory chromatin modifications in breast cancer cells [73], and single-cell analysis of chromatin accessibility performed in a variety of adult mouse tissues, developing mouse mammary glands, and mouse models of breast cancer [74–76]. Furthermore, a variety of “multi-omics” single-cell experiments have emerged, combining transcriptomics, epigenomics, and proteomics analysis, offering hope for future experiments [77, 78]. The combination of single-cell epigenetics and transcriptomics may provide new insights into the role of the SWI/SNF complex in breast cancer. Recognizing and comprehending the presence of cellular heterogeneity in breast cancer is crucial, as it can inform clinical strategies aimed at addressing tumor heterogeneity. By gaining insights into this complex landscape, novel opportunities arise for the development of precise and targeted therapeutics. Consequently, disrupting the overall transcriptional program by targeting specific epigenetic regulators represents a promising avenue to impede the progression of cancer [63].

Combining epigenetic therapy with conventional chemotherapy has great potential in the treatment of cancer, focusing on the SWI/SNF complex could potentially augment the efficacy of current treatment protocols. To fully ascertain this potential, further investigation is required to discern the disparities in SWI/SNF subunit expression between normal and cancer cells [70]. While our understanding of the relationship between SWI/SNF subunit expression is still limited, available literature indicates that disrupting one ATPase can result in the upregulation of the other ATPase, a phenomenon known as functional compensation. This concept poses additional challenges for therapies targeting both ATPases. Furthermore, significant gaps remain in our knowledge regarding how manipulating one subunit affects the expression of other subunits [79], the assembly process of the enzyme complex [48], and the subsequent consequences for both individual subunits and the overall functionality of the enzyme [18]. Additional research is imperative to unravel the complexities of these relationships and comprehend their implications for future therapeutic interventions. The dysregulation of the SWI/SNF complex is multifaceted, making it difficult to identify correlations between single biomarkers and clinical pathological patient characteristics and survival rates [80]. However, this is the beginning of an exciting new research phase, and undoubtedly people will continue to search for other molecules that can inhibit cancer cell proliferation. The real challenge lies in conducting additional studies to ascertain the efficacy and specificity of these molecules in targeting breast cancer patients, extending beyond the confines of cancer cell models cultivated in vitro.

## Data Availability

No datasets were generated or analysed during the current study.

## Abbreviations

5-FU: 5- fluorouracil
ABC: ATP-binding cassette transporter
ARID1A: AT-rich interaction domain 1 A
ARID1B: AT-rich interaction domain 1B
ARID2: AT-rich interaction domain 2
ATP: Adenosine triphosphate
BAF: BRG1/BRM-associated factor
BRCA1: Breast cancer 1
BRD: Bromodomain containing
BRG1: Brahma-related gene 1
BRM: Brahma
CDK4/6: Cyclin-dependent kinase 4/6
CHIP-seq: Chromatin immunoprecipitation- sequence
CRISPR/ Cas9: Clustered regularly interspaced short palindromic repeats/ CRISPR associated protein 9
DNA: Deoxyribo nucleic acid
E2F: E2 transcription factors
EP300: E1A binding protein p300
ER: estrogen receptor
EZH2: Enhancer of zeste homolog 2
GATA3: GATA binding protein 3
GLTSCR1/GLTSCR1L: Glioma tumor suppressor candidate region gene 1/ Glioma tumor suppressor candidate region gene 1-like
HDAC2/6: histone deacetylase 2/6
HER2: Human epidermal growth factor receptor 2
ncBAF: Non-canonical BAF
PARP1: poly (ADP-ribose) polymerase 1
PBAF: Polybromo-associated BAF
PBRM1: Polybromo 1
PI3K/AKT: Phosphatidylinositol 3-kinase/ protein kinase B
PIK3CA: Phosphoinositide-3-kinase, catalytic, alpha polypeptide
PR: Progesterone receptor
PTEN: phosphatase and tensin homolog deleted on chromosome ten
RNA: Ribonucleic Acid
SMARCA2: SWI/SNF related, matrix associated, actin dependent regulator of chromatin, subfamily A, member 2
SMARCA4: SWI/SNF related, matrix associated, actin dependent regulator of chromatin, subfamily A, member 4
SNF2a: Sucrose nonfermenting 2a
SNF2b: Sucrose nonfermenting 2b
SWI/SNF: Switch/sucrose non-fermentable
TGF-β: Transforming growth factor-β
TP53: Tumor protein P53

## References

[1] Villarreal-Garcia V, Estupinan-Jimenez JR, Vivas-Mejia PE, Gonzalez-Villasana V, Vazquez-Guillen JM, Resendez-Perez D (2022). A vicious circle in breast cancer: the interplay between inflammation, reactive oxygen species, and microRNAs. Front Oncol.

[2] Sung H, Ferlay J, Siegel RL, Laversanne M, Soerjomataram I, Jemal A (2021). Global Cancer statistics 2020: GLOBOCAN estimates of incidence and Mortality Worldwide for 36 cancers in 185 countries. CA Cancer J Clin.

[3] Nickerson JA, Wu Q, Imbalzano AN (2017). Mammalian SWI/SNF enzymes and the epigenetics of Tumor Cell metabolic reprogramming. Front Oncol.

[4] Wang X, Roberts CW (2014). CARMA: CARM1 methylation of SWI/SNF in breast cancer. Cancer Cell.

[5] Sethuraman A, Brown M, Seagroves TN, Wu ZH, Pfeffer LM, Fan M (2016). SMARCE1 regulates metastatic potential of breast cancer cells through the HIF1A/PTK2 pathway. Breast Cancer Res.

[6] Weissman B, Knudsen KE (2009). Hijacking the chromatin remodeling machinery: impact of SWI/SNF perturbations in cancer. Cancer Res.

[7] Saha A, Wittmeyer J, Cairns BR (2006). Chromatin remodelling: the industrial revolution of DNA around histones. Nat Rev Mol Cell Bio.

[8] Luchini C, Veronese N, Solmi M, Cho H, Kim JH, Chou A (2015). Prognostic role and implications of mutation status of tumor suppressor gene ARID1A in cancer: a systematic review and meta-analysis. Oncotarget.

[9] Wu JI, Lessard J, Crabtree GR (2009). Understanding the words of chromatin regulation. Cell.

[10] Michel BC, D’Avino AR, Cassel SH, Mashtalir N, McKenzie ZM, McBride MJ (2018). A non-canonical SWI/SNF complex is a synthetic lethal target in cancers driven by BAF complex perturbation. Nat Cell Biol.

[11] Pan J, Meyers RM, Michel BC, Mashtalir N, Sizemore AE, Wells JN (2018). Interrogation of mammalian protein complex structure, function, and membership using genome-scale fitness screens. Cell Syst.

[12] Alpsoy A, Dykhuizen EC (2018). Glioma tumor suppressor candidate region gene 1 (GLTSCR1) and its paralog GLTSCR1-like form SWI/SNF chromatin remodeling subcomplexes. J Biol Chem.

[13] Roberts CW, Orkin SH (2004). The SWI/SNF complex–chromatin and cancer. Nat Rev Cancer.

[14] Biegel JA, Busse TM, Weissman BE (2014). SWI/SNF chromatin remodeling complexes and cancer. Am J Med Genet C Semin Med Genet.

[15] Feinberg AP (2014). Epigenetic stochasticity, nuclear structure and cancer: the implications for medicine. J Intern Med.

[16] Wilson BG, Roberts CW (2011). SWI/SNF nucleosome remodellers and cancer. Nat Rev Cancer.

[17] Varela I, Tarpey P, Raine K, Huang D, Ong CK, Stephens P (2011). Exome sequencing identifies frequent mutation of the SWI/SNF complex gene PBRM1 in renal carcinoma. Nature.

[18] Wu Q, Lian JB, Stein JL, Stein GS, Nickerson JA, Imbalzano AN (2017). The BRG1 ATPase of human SWI/SNF chromatin remodeling enzymes as a driver of cancer. Epigenomics.

[19] Cui Y, Bai X, Niu M, Qin Y, Zhang X, Pang D (2019). Upregulated expression of AT-rich interactive domain-containing protein 1B predicts poor prognosis in patients with triple-negative breast cancer. Oncol Lett.

[20] Kadoch C, Hargreaves DC, Hodges C, Elias L, Ho L, Ranish J (2013). Proteomic and bioinformatic analysis of mammalian SWI/SNF complexes identifies extensive roles in human malignancy. Nat Genet.

[21] Wu JN, Roberts CW (2013). ARID1A mutations in cancer: another epigenetic tumor suppressor?. Cancer Discov.

[22] Davó-Martínez C, Helfricht A, Ribeiro-Silva C, Raams A, Tresini M, Uruci S (2023). Different SWI/SNF complexes coordinately promote R-loop- and RAD52-dependent transcription-coupled homologous recombination. Nucleic Acids Res.

[23] Jones S, Stransky N, McCord CL, Cerami E, Lagowski J, Kelly D (2014). Genomic analyses of gynaecologic carcinosarcomas reveal frequent mutations in chromatin remodelling genes. Nat Commun.

[24] Mao TL, Shih Ie M (2013). The roles of ARID1A in gynecologic cancer. J Gynecol Oncol.

[25] Samartzis EP, Noske A, Dedes KJ, Fink D, Imesch P (2013). ARID1A mutations and PI3K/AKT pathway alterations in endometriosis and endometriosis-associated ovarian carcinomas. Int J Mol Sci.

[26] Yates LR, Knappskog S, Wedge D, Farmery JHR, Gonzalez S, Martincorena I (2017). Genomic evolution of breast Cancer metastasis and relapse. Cancer Cell.

[27] Zhang XW, Zhang YX, Zhao JY, Wu YJ, Zhang N, Shen WJ (2023). ARID1A mutations in cancer development: mechanism and therapy. Carcinogenesis.

[28] Schwartz CJ, Pareja F, da Silva EM, Selenica P, Ross DS, Weigelt B (2021). Histologic and genomic features of breast cancers with alterations affecting the SWI/SNF (SMARC) genes. Mod Pathol.

[29] Sun XX, Wang SC, Wei YL, Luo X, Jia YM, Li L (2017). Has context-dependent oncogenic and tumor suppressor functions in Liver Cancer. Cancer Cell.

[30] Szpon L, Agrawal A, Jelen M, Lipinski A, Rudnicki J, Makuch S (2020). ARID1A/BAF250a is significantly overexpressed in primary invasive breast cancer. Transl Cancer Res.

[31] Guo X, Zhang Y, Mayakonda A, Madan V, Ding LW, Lin LH (2018). ARID1A and CEBPalpha cooperatively inhibit UCA1 transcription in breast cancer. Oncogene.

[32] Xu G, Chhangawala S, Cocco E, Razavi P, Cai Y, Otto JE (2020). ARID1A determines luminal identity and therapeutic response in estrogen-receptor-positive breast cancer. Nat Genet.

[33] Suryo Rahmanto Y, Shen W, Shi X, Chen X, Yu Y, Yu ZC (2020). Inactivation of Arid1a in the endometrium is associated with endometrioid tumorigenesis through transcriptional reprogramming. Nat Commun.

[34] Allo G, Bernardini MQ, Wu RC, Shih Ie M, Kalloger S, Pollett A (2014). ARID1A loss correlates with mismatch repair deficiency and intact p53 expression in high-grade endometrial carcinomas. Mod Pathol.

[35] Pimienta LM, Sanchez JCS, Hernandez I (2023). ARID1A alterations and their clinical significance in breast Cancer. Univ Med.

[36] Jdeed S, Lengyel M, Uray IP (2022). Redistribution of the SWI/SNF Complex Dictates Coordinated Transcriptional Control over epithelial-mesenchymal transition of normal breast cells through TGF-beta signaling. Cells..

[37] Sasaki M, Ogiwara H (2020). Synthetic lethal therapy based on targeting the vulnerability of SWI/SNF chromatin remodeling complex-deficient cancers. Cancer Sci.

[38] Fang BL (2014). Development of synthetic lethality anticancer therapeutics. J Med Chem.

[39] Zhang X, Zhang Y, Yang Y, Niu M, Sun S, Ji H (2012). Frequent low expression of chromatin remodeling gene ARID1A in breast cancer and its clinical significance. Cancer Epidemiol.

[40] Shen J, Peng Y, Wei L, Zhang W, Yang L, Lan L (2015). ARID1A Deficiency impairs the DNA damage checkpoint and sensitizes cells to PARP inhibitors. Cancer Discov.

[41] Cheng X, Zhao JX, Dong F, Cao XC (2021). ARID1A mutation in metastatic breast Cancer: a potential therapeutic target. Front Oncol.

[42] Nagl NG, Wang X, Patsialou A, Van Scoy M, Moran E (2007). Distinct mammalian SWI/SNF chromatin remodeling complexes with opposing roles in cell-cycle control. EMBO J.

[43] Shao F, Guo T, Chua PJ, Tang L, Thike AA, Tan PH (2015). Clinicopathological significance of ARID1B in breast invasive ductal carcinoma. Histopathology.

[44] Yan Z, Wang Z, Sharova L, Sharov AA, Ling C, Piao Y (2008). BAF250B-associated SWI/SNF chromatin-remodeling complex is required to maintain undifferentiated mouse embryonic stem cells. Stem Cells.

[45] Gao XL, Tate P, Hu P, Tjian R, Skarnes WC, Wang Z (2008). ES cell pluripotency and germ-layer formation require the SWI/SNF chromatin remodeling component BAF250a. P Natl Acad Sci USA.

[46] Helming KC, Wang X, Wilson BG, Vazquez F, Haswell JR, Manchester HE (2014). ARID1B is a specific vulnerability in ARID1A-mutant cancers. Nat Med.

[47] Xu S, Tang C (2021). The role of ARID1A in tumors: Tumor initiation or tumor suppression?. Front Oncol.

[48] Mashtalir N, D’Avino AR, Michel BC, Luo J, Pan J, Otto JE (2018). Modular Organization and Assembly of SWI/SNF family chromatin remodeling complexes. Cell.

[49] Stephens PJ, Tarpey PS, Davies H, Van Loo P, Greenman C, Wedge DC (2012). The landscape of cancer genes and mutational processes in breast cancer. Nature.

[50] Hopson S, Thompson MJ (2017). BAF180: its roles in DNA repair and consequences in Cancer. ACS Chem Biol.

[51] Hagiwara M, Fushimi A, Yamashita N, Bhattacharya A, Rajabi H, Long MD (2021). MUC1-C activates the PBAF chromatin remodeling complex in integrating redox balance with progression of human prostate cancer stem cells. Oncogene.

[52] Yamashita N, Morimoto Y, Fushimi A, Ahmad R, Bhattacharya A, Daimon T (2023). MUC1-C dictates PBRM1-Mediated chronic induction of Interferon Signaling, DNA damage resistance, and Immunosuppression in Triple-negative breast Cancer. Mol Cancer Res.

[53] Xia W, Nagase S, Montia AG, Kalachikov SM, Keniry M, Su T (2008). BAF180 is a critical regulator of p21 induction and a tumor suppressor mutated in breast cancer. Cancer Res.

[54] Mo D, Li C, Liang J, Shi Q, Su N, Luo S (2015). Low PBRM1 identifies tumor progression and poor prognosis in breast cancer. Int J Clin Exp Pathol.

[55] Miao D, Margolis CA, Gao W, Voss MH, Li W, Martini DJ (2018). Genomic correlates of response to immune checkpoint therapies in clear cell renal cell carcinoma. Science.

[56] Chabanon RM, Morel D, Eychenne T, Colmet-Daage L, Bajrami I, Dorvault N (2021). PBRM1 Deficiency confers synthetic lethality to DNA repair inhibitors in Cancer. Cancer Res.

[57] Pan D, Kobayashi A, Jiang P, Ferrari de Andrade L, Tay RE, Luoma AM (2018). A major chromatin regulator determines resistance of tumor cells to T cell-mediated killing. Science.

[58] Hodges C, Kirkland JG, Crabtree GR (2016). The many roles of BAF (mSWI/SNF) and PBAF complexes in Cancer. Cold Spring Harb Perspect Med..

[59] Zhang J, Hou S, You Z, Li G, Xu S, Li X (2021). Expression and prognostic values of ARID family members in breast cancer. Aging.

[60] Smith JJ, Xiao Y, Parsan N, Medwig-Kinney TN, Martinez MAQ, Moore FEQ (2022). The SWI/SNF chromatin remodeling assemblies BAF and PBAF differentially regulate cell cycle exit and cellular invasion in vivo. PLoS Genet.

[61] Hoffman GR, Rahal R, Buxton F, Xiang K, McAllister G, Frias E (2014). Functional epigenetics approach identifies BRM/SMARCA2 as a critical synthetic lethal target in BRG1-deficient cancers. Proc Natl Acad Sci U S A.

[62] Wu JI (2012). Diverse functions of ATP-dependent chromatin remodeling complexes in development and cancer. Acta Biochim Biophys Sin (Shanghai).

[63] Wu Q, Madany P, Akech J, Dobson JR, Douthwright S, Browne G (2015). The SWI/SNF ATPases are required for Triple negative breast Cancer Cell Proliferation. J Cell Physiol.

[64] Shaykevich A, Silverman I, Bandyopadhyaya G, Maitra R (2023). BRG1: promoter or suppressor of cancer? The outcome of BRG1's interaction with specific cellular pathways. Int J Mol Sci.

[65] Bai J, Mei P, Zhang C, Chen F, Li C, Pan Z (2013). BRG1 is a prognostic marker and potential therapeutic target in human breast cancer. PLoS ONE.

[66] Sobczak M, Pitt AR, Spickett CM, Robaszkiewicz A (2019). PARP1 co-regulates EP300-BRG1-dependent transcription of genes involved in breast cancer cell proliferation and DNA repair. Cancers (Basel).

[67] Sobczak M, Pietrzak J, Ploszaj T, Robaszkiewicz A (2020). BRG1 activates proliferation and transcription of cell cycle-dependent genes in breast cancer cells. Cancers.

[68] Dietrich N, Hoffman JA, Archer TK (2020). BAF Complexes and the glucocorticoid receptor in breast cancers. Curr Opin Endocr Metab Res.

[69] Wu Q, Madany P, Dobson JR, Schnabl JM, Sharma S, Smith TC (2016). The BRG1 chromatin remodeling enzyme links cancer cell metabolism and proliferation. Oncotarget.

[70] Wu Q, Sharma S, Cui H, LeBlanc SE, Zhang H, Muthuswami R (2016). Targeting the chromatin remodeling enzyme BRG1 increases the efficacy of chemotherapy drugs in breast cancer cells. Oncotarget.

[71] Dubey R, Lebensohn AM, Bahrami-Nejad Z, Marceau C, Champion M, Gevaert O (2016). Chromatin-remodeling complex SWI/SNF controls Multidrug Resistance by Transcriptionally regulating the drug Efflux Pump ABCB1. Cancer Res.

[72] Reddy D, Bhattacharya S, Workman JL (2023). mis)-Targeting of SWI/SNF complex(es) in cancer. Cancer Metast Rev.

[73] Grosselin K, Durand A, Marsolier J, Poitou A, Marangoni E, Nemati F (2019). High-throughput single-cell ChIP-seq identifies heterogeneity of chromatin states in breast cancer. Nat Genet.

[74] Cusanovich DA, Hill AJ, Aghamirzaie D, Daza RM, Pliner HA, Berletch JB (2018). A single-cell atlas of in vivo mammalian chromatin accessibility. Cell.

[75] Chung CY, Ma Z, Dravis C, Preissl S, Poirion O, Luna G (2019). Single-cell chromatin analysis of mammary Gland Development reveals cell-state transcriptional regulators and Lineage relationships. Cell Rep.

[76] Chen X, Miragaia RJ, Natarajan KN, Teichmann SA (2018). A rapid and robust method for single cell chromatin accessibility profiling. Nat Commun.

[77] Reyes M, Billman K, Hacohen N, Blainey PC (2019). Simultaneous profiling of gene expression and chromatin accessibility in single cells. Adv Biosyst.

[78] Hu Y, An Q, Sheu K, Trejo B, Fan S, Guo Y (2018). Single cell Multi-omics Technology: methodology and application. Front Cell Dev Biol.

[79] Otto JE, Ursu O, Wu AP, Winter EB, Cuoco MS, Ma S (2023). Structural and functional properties of mSWI/SNF chromatin remodeling complexes revealed through single-cell perturbation screens. Mol Cell.

[80] Pries K, Kruger S, Heckl S, Behrens HM, Rocken C (2023). SMARCA4 and SMARCE1 in gastric cancer: correlation with ARID1A, and microsatellite stability, and SMARCE1/ERBB2 co-amplification. Cancer Med-Us.

